# 660. Accurately Attributing Infections to the Source Using a Wound Care Template

**DOI:** 10.1093/jbcr/irag033.497

**Published:** 2026-04-06

**Authors:** Michelle Kaleita, Janet A Popp, Austin K Saunders, Andrea Munden, Michelle L Robinson Rener

**Affiliations:** University of Florida Health Shands Burn Center, Gainesville, FL; University of Florida Health Shands Burn Center, Gainesville, FL; University of Florida Health Shands Burn Center, Gainesville, FL; University of Florida, Gainesville, FL; University of Florida Health, Gainesville, FL

## Abstract

**Introduction:**

Sepsis is a complication that burned patients encounter which can lead to a high chance of mortality. Burn wound infections increase the risk of sepsis. Indwelling lines are often necessary in treatment and can also contribute to bloodstream infections (BSIs). NHSN (National Healthcare Safety Network) has specific signs and symptoms for attributing positive cultures to various sources. There are additional criteria depending upon the type of graft (e.g., autograft versus allograft).

Daily wound assessments help with early identification of changes and potential infections. Due to staff turnover and lack of expertise, documentation became inadequate. Pictures in the electronic medical record served as a primary source of communication. Written documentation describing wound changes is required by NHSN for BSI attribution. In Aug 2024, a central line related BSI (CLABSI) was attributed due to the lack of adequate wound descriptors despite multiple surgeries and matching wound cultures.

**Methods:**

The EPIC burn wound assessment flowsheet was not specific enough to meet NHSN and provider expectations. Several templates were explored with input from the providers, the Unit Practice Council, and Infection Control Practitioner (ICP). In Oct 2024, a modified version of a wound care note template that is used at another major burn facility was implemented.

Staff were educated on adequate wound assessment. Notes were evaluated by the Clinical Leader (CL) and ICP to ensure that the content accurately described the wounds while meeting the criteria required by NHSN. During weekly line rounds and chart reviews, staff interactions included feedback on wound notes and time for questions.

**Results:**

A retrospective review of 17 positive BSIs from Jan 2024-Sept 2024 was conducted. Two CLABSIs were attributed to lack of wound documentation. Four of the 17 events had adequate documentation attributing the BSI to the burn wounds. From Oct 2024-Aug 2025 one true CLABSI was attributed, as the documentation on the wound note template supported that this was not due to the wounds. Of the remaining 35 events, 20 were attributed to the burn wounds due to adequate documentation.

**Conclusions:**

A wound note template provides a consistent location for all nursing staff to adequately provide descriptors of wounds. Orientation and ongoing education on proper documentation and terminology is necessary to communicate across the multidiscipline team. Other departments are looking to adopt a similar template to ensure that their documentation meets or exceeds standards.

**Applicability of Research to Practice:**

The prompts of a wound note template have been shown to demonstrate consistency and accuracy.

Accurate documentation facilitates attributing infections to the proper source which can lead to line salvage, better antibiotic stewardship, and better patient outcomes.

Improved wound care documentation broadens the ability to accurately attribute BSIs to the source.

**Funding for the study:**

N/A.

